# Dose-Dependent ATP Depletion and Cancer Cell Death following Calcium Electroporation, Relative Effect of Calcium Concentration and Electric Field Strength

**DOI:** 10.1371/journal.pone.0122973

**Published:** 2015-04-08

**Authors:** Emilie Louise Hansen, Esin Bengisu Sozer, Stefania Romeo, Stine Krog Frandsen, P. Thomas Vernier, Julie Gehl

**Affiliations:** 1 Center for Experimental Drug and Gene Electrotransfer, Department of Oncology, Copenhagen University Hospital Herlev, Copenhagen, Denmark; 2 Frank Reidy Research Center for Bioelectrics, Old Dominion University, Norfolk, Virginia, United States of America; 3 Institute for Electromagnetic Sensing of the Environment (IREA), Italian National Research Council, Naples, Italy; University of California at Berkeley, UNITED STATES

## Abstract

**Background:**

Electroporation, a method for increasing the permeability of membranes to ions and small molecules, is used in the clinic with chemotherapeutic drugs for cancer treatment (electrochemotherapy). Electroporation with calcium causes ATP (adenosine triphosphate) depletion and cancer cell death and could be a novel cancer treatment. This study aims at understanding the relationship between applied electric field, calcium concentration, ATP depletion and efficacy.

**Methods:**

In three human cell lines — H69 (small-cell lung cancer), SW780 (bladder cancer), and U937 (leukaemia), viability was determined after treatment with 1, 3, or 5 mM calcium and eight 99 μs pulses with 0.8, 1.0, 1.2, 1.4 or 1.6 kV/cm. Fitting analysis was applied to quantify the cell-killing efficacy in presence of calcium. Post-treatment intracellular ATP was measured in H69 and SW780 cells. Post-treatment intracellular ATP was observed with fluorescence confocal microscopy of quinacrine-labelled U937 cells.

**Results:**

Both H69 and SW780 cells showed dose-dependent (calcium concentration and electric field) decrease in intracellular ATP (p<0.05) and reduced viability. The 50% effective cell kill was found at 3.71 kV/cm (H69) and 3.28 kV/cm (SW780), reduced to 1.40 and 1.15 kV/cm (respectively) with 1 mM calcium (lower EC50 for higher calcium concentrations). Quinacrine fluorescence intensity of calcium-electroporated U937 cells was one third lower than in controls (p<0.0001).

**Conclusions:**

Calcium electroporation dose-dependently reduced cell survival and intracellular ATP. Increasing extracellular calcium allows the use of a lower electric field.

**General Significance:**

This study supports the use of calcium electroporation for treatment of cancer and possibly lowering the applied electric field in future trials.

## Introduction

Electroporation is a method used to generate increased porousness of cell membranes by applying permeabilising electric pulses [1]. The method can be used to induce passage of otherwise non-permeating drugs [2–5] or ions such as calcium [6,7] into cells through the cell membrane [8].

Reversible electroporation, where cell membranes reseal after transient permeabilisation, is used in the clinic in combination with chemotherapeutic drugs for local treatment of malignant tumours (electrochemotherapy) [9–11]. Substituting calcium for chemotherapeutic agents in this procedure (calcium electroporation) could be a novel, efficient and inexpensive cancer treatment [6].

It is of great clinical interest to explore the possibility of minimizing the electric field magnitude during treatment, both in the avoidance of muscle contractions and in the development of electrodes that can treat deep-seated tumours. Exposing cells to split-dose electroporation has been shown to increase cell sensitivity to electrochemotherapy, a phenomenon called electrosensitation [12]. Our study aims to investigate a possible increase in sensitivity also to irreversible electroporation treatment when increasing extracellular calcium. Irreversible electroporation (IRE) [13] is another electroporation based therapy, where stronger electric fields are used to kill cancer cells through irreversible membrane permeabilisation. Increasing electrode therapeutic range by reducing the electric field is a particular concern in IRE, as well as other electroporation based therapies [14]. For IRE, adding calcium might increase the treatment radius and efficacy.

Calcium is a ubiquitous second messenger involved in numerous cellular processes including controlled necrosis (necroptosis) [15–18]. The concentration gradient for calcium across the cell membrane is tightly regulated in order to maintain cellular homeostasis. In normal cells the intracellular concentration of ionized calcium is maintained around 100 nM, substantially lower than the extracellular concentration, which typically exceeds 1 mM [16,19]. The gradient is maintained through numerous membrane pumps, such as Ca^2+^-ATPases, that consume ATP (adenosine triphosphate) for energy to either expel excess calcium from the cell or transfer it into cellular compartments such as the endoplasmatic reticulum or mitochondria [20–22].

We have shown previously that cellular ATP levels drop following calcium electroporation [6]. When a dramatic increase in intracellular calcium occurs, potentially by calcium electroporation, an increased activity of Ca^2+^-ATPases hastily consumes cellular ATP [19]. An increase in calcium also affects the mitochondria leading to loss of ATP production [20]. Furthermore the creation of transient pores in the plasma membrane may cause leakage of ATP out of the cell [23]. Overall, cellular ATP diminishes when a toxic level of calcium is induced into the cell, possibly through these mechanisms. This ATP depletion may contribute to initiation of cell death [6,24].

Calcium electroporation is comparable in effectiveness to electrochemotherapy *in vitro* [7] and is currently undergoing the first clinical trial in a randomized double blinded phase II non-inferiority study (ClinicalTrials.gov-ID NCT01941901). More knowledge regarding the mechanism of calcium electroporation is needed to further understand and exploit this antitumor modality.

To investigate the relation between calcium concentration and pulsed electric fields in obtaining ATP depletion and sufficient cell kill *in vitro*, we used cell lines known to have different responsiveness to calcium electroporation treatment *in vivo*: H69 human small-cell lung cancer with high sensitivity (77–100% necrosis in tumours 6 days after calcium electroporation; [9]) and SW780 human bladder cancer cell line with low sensitivity (1–60% necrosis in tumours 6 days after calcium electroporation; unpublished data). We also explored the short term effects of calcium electroporation on the ATP content of a human leukaemia cell line (U937) using confocal imaging with the fluorescent marker quinacrine.

This study investigates the relationship between applied electric field, calcium concentration, cell line sensitivity and the effect on ATP content and survival.

## Materials and Methods

Three human cell lines were used for experiments. Experiments analysing ATP content and viability performed at Copenhagen University Hospital Herlev used H69 human small-cell lung cancer and SW780 human bladder cancer cell lines. The U937 human leukaemia cell line was used for experiments at the Frank Reidy Research Center for Bioelectrics.

### Cell culturing

The H69 human small-cell lung cancer cell line stably transfected with enhanced green fluorescent protein (EGFP) regulated by the cytomegalovirus promotor [25] was kindly provided by the Department of Radiation Biology, Copenhagen University Hospital. The SW780 human bladder transitional cell carcinoma was kindly provided by Dr. Lars Dyrskjøt Andersen, Department of Molecular Medicine, Aarhus University Hospital [26]. The U937 human histiocytic lymphoma monocyte cell line (ATCC CRL-1593.2) was kindly provided by Olga Pakhomova, Frank Reidy Research Center for Bioelectrics, Old Dominion University.

All cell lines were maintained in RPMI 1640 culture medium containing 10% foetal calf serum (Gibco, Invitrogen), 100 U/ml penicillin and 100 μg/ml streptomycin at 37°C and 5% CO_2_.

### Electroporation protocol

Cells in HEPES buffer (10 mM HEPES (Lonza), 250 mM sucrose, 1 mM MgCl_2_ in sterile water) with final concentration 6.1 × 10^6^ cells/ml, were chilled on ice for 5 min. For calcium electroporation (CaEP), 20 μl HEPES buffer containing CaCl_2_ was added to electroporation cuvettes with 4 mm electrode separation (Molecular BioProducts Inc., San Diego, CA, USA) to yield a final concentration of 0 mM, 1 mM, 3 mM, or 5 mM CaCl_2_ after adding 180 μl of cell suspension. The cuvettes were kept on ice for 5 min prior to electroporation (EP). EP consisted of 8 pulses of 99 μs with 0.8 kV/cm, 1.0 kV/cm, 1.2 kV/cm, 1.4 kV/cm or 1.6 kV/cm respectively, delivered at a frequency of 1 Hz using a BTX T820 square wave electroporator (BTX Harvard Apparatus). After 20 minutes incubation at 37°C and 5% CO_2_, cells were diluted in 5 ml culture medium (final concentration 2.4 × 10^5^ cells/ml).

### ATP assay

Cellular ATP level was measured in H69 and SW780 cells treated with calcium with or without electroporation using ATP luminescence assay (Enliten ATP Assay; Promega) after cell lysis. For ATP assay 11.5 × 10^4^ cells/ml H69 cells or 4.6 × 10^4^ cells/ml SW780 cells in 100 μl culture medium were in 96-well plates in accordance with initial optimization.

Cells were treated according to the electroporation protocol. Cells electroporated with HEPES buffer, non-electroporated cells with calcium, and untreated cells were used as controls. Cells thrice thawed and frozen, then sonicated (Ultrasonic Bath Branson 1200), were used as control (dead cells). After centrifugation of the 96-well plates supernatant was removed. Cellular ATP content 1, 2, 4, and 8 hours after treatment was determined by lysing the cells (30 min) then adding 100 μl rl/l reagent and measuring light emission using a luminometer (LUMIstar, Ramcon).

### MTS Viability assay

Viability of H69 and SW780 cells treated with calcium with or without electroporation was assessed using MTS (3-(4,5-dimethylthiazol-2-yl)-5-(carboxymethophenyl)-2-(4-sulfonyl)-2H-tetrazolium) assay (Cell Titer 96 Aqueous Non-Radioactive Cell Proliferation Assay; Promega). MTS assay is a colorimetric method that estimates the rate of metabolism in viable cells. After treatment 100 μl cell suspension (2.4 × 10^5^ cells/ml) was seeded in 96-well plates. After 24 hours incubation 20 μl MTS/PMS reagent was added to each well (final concentrations of 333 μg/ml MTS and 25 μM PMS). After 1 hour of incubation at 37°C and 5% CO_2_ cell viability was assessed by measuring optical density (OD) using Multiscan-Ascent ELISA reader (ThermoLabsystems, Finland) at 490 nm with background measurements at 690 nm.

### Fitting relations between experimental parameters and cell survival data

The data analysis was performed using MATLAB (Natick, MA, USA) software. The cell survival (expressed as percentage of control) of H69 and SW780 cell lines at 24 h after treatment was plotted against the electric field amplitude at each calcium concentration considered. The best fitting curve was then derived. From the fitting curves, the electric field strength (E_50_) required to kill at least 50% of cells at each calcium concentration was extracted, in order to quantify efficiency of cell killing of electroporation in absence or in presence of calcium.

### ATP imaging

Using confocal scanning imaging with a Leica TCS SP8 microscope, ATP content after electroporation was assessed in U937 cells using the fluorescent dye quinacrine (Sigma-Aldrich, USA), a non-specific marker with high affinity for adenine nucleotides [15]. Four conditions were explored: Cells treated with 3 mM external calcium; cells exposed to electroporating pulses (1 kV/cm, 8 × 99 μs, 1 Hz); cells exposed to both 3 mM external calcium and electroporating pulses; and untreated control samples. Cells were incubated at 37°C for 30 minutes in growth medium containing 10 μM quinacrine, followed by the electroporation protocol described in section 2.2. (Same cell concentration used but cooling excluded and cells were electroporated in 1 mm cuvettes in presence of 10 μM propidium iodide). Immediately after electroporation cells were transferred respectively to cover glass chambers for imaging containing RPMI 1640 medium, 1.6 μM quinacrine and 1.25 μM propidium iodide (Molecular Probes, USA; an indicator of membrane permeabilisation) with 1:6 dilution of both cells and dyes (cell concentration 1 × 10^6^ cells/ml) and to 96-well plates for viability assay. We imaged the quinacrine fluorescence 15 minutes after treatment. The fluorescence intensity was measured in 9 images per condition. In each image, fluorescence of twenty randomly selected cells was integrated and averaged using MATLAB software (180 cells total).

### Resazurin viability assay

Viability of U937 cells was assessed using resazurin assay (Life Technologies, USA) conducted 24 hours after exposure. Ten microliters reagent (Prestoblue, Molecular Probes) was added to each well containing 90 μl cell suspension (cell concentration 2.4 × 10^5^ cells/ml). After 1 hour of incubation at 37°C and 5% CO_2_, cell viability was assessed by measuring fluorescence using Synergy 2 microplate reader (BioTek Instruments, USA).

### Statistical analyses

Differences in ATP luminescence intensity between groups tested with ATP luminescence assay were validated and analysed with an exponential decrease model with Bonferroni correction using SAS software (version 9.2). Luminescence values were log transformed before analysis. Two-way ANOVA (Analysis of variance) with Bonferroni correction was used to assess differences in viability between groups tested with MTS proliferation assay also using SAS 9.2 software. T-test was used for assessment of differences between groups tested with resazurin viability assay and for imaged groups analysed with MATLAB software.

## Results

### Cellular ATP and viability after calcium electroporation

#### Calcium only (no electroporation)

**Fig 1:**
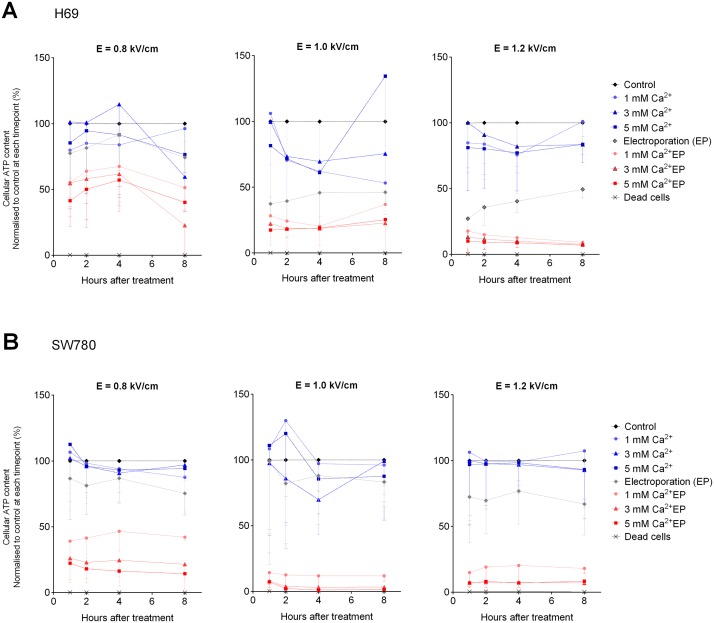
Cellular ATP content in H69 and SW780 cells after calcium electroporation. Cellular ATP levels determined 1, 2, 4, and 8 hours after calcium electroporation treatment of two human cell lines (H69, a small cell lung cancer cell line (**A**); and SW780, a bladder cancer cell line (**B**)). Extracellular calcium concentrations of 0 mM, 1 mM, 3 mM, or 5 mM and applied electric field of 0.8 kV/cm, 1.0 kV/cm, or 1.2 kV/cm. Results are illustrated as percentage of control (no electroporation, no added calcium). Normalized to control at each time point (%), mean—S.D., n = 6.

**Fig 2:**
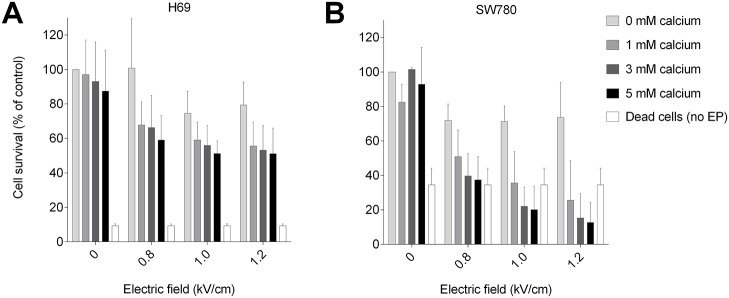
Viability of H69 and SW780 cells after calcium electroporation. Viability, assessed using MTS assay, of H69, a human small cell lung cancer cell line (**A**); and SW780, a human bladder cancer cell line (**B**) 24 hours after treatment with calcium electroporation. Final extracellular calcium concentrations of 0 mM, 1 mM, 3 mM, or 5 mM and applied electric field of either 0.8 kV/cm, 1.0 kV/cm, or 1.2 kV/cm. Cells thrice thawed and frozen, then sonicated were used as control (dead cells). Results are illustrated as percentage of control (no electroporation, no added calcium), electroporation (EP), mean + S.D., n = 6.

Exposing cells to increasing extracellular concentrations of calcium in the absence of electroporation had no apparent effect on cellular ATP level or viability compared to controls in either of the two cell lines (Figs 1A and 1B, 2A and 2B).

#### Electroporation only (no calcium)

Increasing the electric field had no significant effect on the ATP content of H69 cells, when exposing them to electroporation alone (Fig 1A). ATP levels in electroporated-only H69 samples increased over time during incubation as previously observed [6]. There was no significant decline in viability of H69 cells treated with pulsed electric field alone (Fig 2A).

Increasing the electric field from 0.8 to 1.2 kV/cm did not have a significant effect on the ATP content of SW780 cells, when exposing them to electroporation alone (Fig 1B). As shown in Fig 2B, there was a 28% decline in viability of SW780 cells treated with 0.8 kV/cm electric field indicating that electroporation alone caused cell death, although not statistically significant.

#### Calcium electroporation—H69 cell ATP

ATP levels decreased in H69 cells with increasing calcium concentrations and increasing electric field (Fig 1A). Calcium electroporation using 0.8 kV/cm significantly decreased the intracellular ATP level when combined with 5 mM calcium compared with untreated controls (p<0.05). When using 1.0 kV/cm and 1.2 kV/cm, all tested calcium concentrations (1, 3, and 5 mM) significantly decreased the intracellular ATP level compared with untreated controls and calcium alone (p<0.001). Increasing the electric field from 0.8 to 1.0 kV/cm in calcium electroporation with 1 mM calcium significantly decreased the ATP level (p<0.05). Further increasing the field from 1.0 to 1.2 kV/cm had no additional effect on ATP level.

#### Calcium electroporation—H69 cell viability

As seen in Fig 2A, calcium electroporation caused significant decrease in H69 cell viability for all tested doses exceeding 1 mM calcium with 0.8 kV/cm (p<0.05). Further increases in electric field or calcium concentration did not have a significant additional effect on H69 viability (Fig 2A).

#### Calcium electroporation—SW780 cell ATP

**Fig 3:**
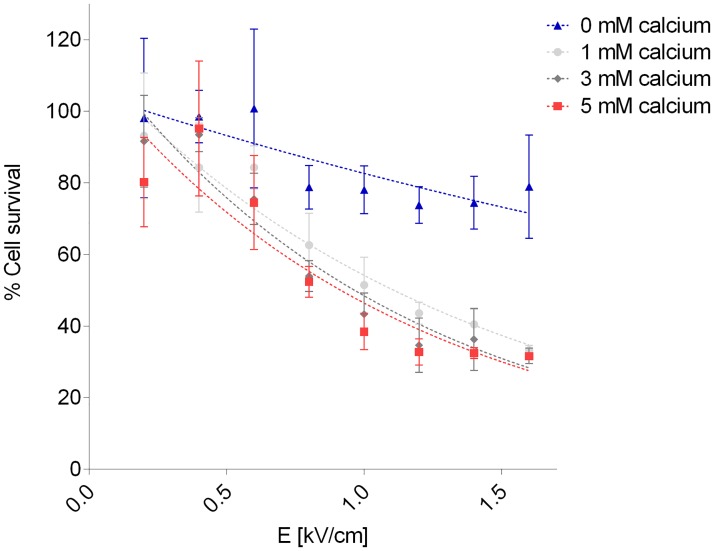
SW780 viability as a function of pulsed electric field and extracellular calcium concentration. Cell survival (%) versus electric field (E) at calcium electroporation using 0, 1, 3, or 5mM calcium in SW780 human bladder cancer cells assessed using MTS assay 24 hours after treatment. Electric field amplitude of 0.8 kV/cm, 1.0 kV/cm, 1.2 kV/cm, 1.4 kV/cm, or 1.6 kV/cm was applied. Fitting curves were derived using MATLAB software. Results are illustrated as percentage of control (no electroporation, no added calcium), mean ± S.D., n = 6.

As shown in Fig 1B, 1, 3 or 5 mM calcium electroporation significantly decreased ATP levels in all pulse conditions compared with untreated controls as well as calcium alone (p<0.05). Increasing the electric field from 0.8 to 1.0 kV/cm, and from 1.0 to 1.2 kV/cm in treatment with 1 mM calcium electroporation significantly decreased the ATP level (p<0.0001). Adding calcium in the electroporation procedure significantly decreased ATP in all pulse conditions (p<0.0001).

#### Calcium electroporation—SW780 cell viability

As schematically shown in Fig 2B, calcium electroporation caused significant decrease in SW780 cell viability for all tested doses exceeding 1 mM calcium with 0.8 kV/cm (p<0.0001) (Fig 2B). Increasing the electric field from 0.8 to 1.2 kV/cm significantly affected SW780 viability in the presence of calcium (p<0.001). Maximum SW780 cell kill required 1.2 kV/cm and 5 mM calcium led to lower values in the MTA assay than freezing and thawing the cells thrice with subsequent sonication (p<0.001).

### Fitting analysis of cell survival data

After analysis with MATLAB curve fitting tool, we found that the best fitting function for the relation between cell survival after 24 hours and the electric field amplitude at fixed calcium concentrations, was a two-parameter exponential function, *y(x) = a·exp(–b·x)* where *x* is the electric field amplitude and *y(x)* is the percentage of cell survival. Results on SW780 cells are reported in Fig 3. The E_50_ parameter at each calcium concentration considered was extracted using the found function. The results, schematically reported in Table 1 for both the H69 and the SW780 cell line, allowed us to quantify the increased cell killing efficiency of electroporation in the presence of calcium. In both cell lines, and for all the calcium concentrations tested, the electric field required to halve cell survival at 24 hours after treatment in presence of extracellular calcium was two- to three times lower than in the case of electroporation alone. Different responses were obtained between the two cell lines. The H69 cell line required higher electric field amplitude (around 20%) than SW780 cells to obtain a 50% reduction in cell viability, with an extracellular concentration of calcium chloride from 1 to 5 mM. The SW780 cell line may be more sensitive to treatment with low doses of calcium than H69 cells *in vitro*.

**Table 1:** SW780 cell sensitivity to electric field with increased extracellular calcium chloride concentrations.

H69		SW780	
[CaCl_2_]	E_50_	[CaCl_2_]	E_50_
0 mM	3.7 kV/cm	0 mM	3.3 kV/cm
1 mM	1.4 kV/cm	1 mM	1.1 kV/cm
3 mM	1.2 kV/cm	3 mM	0.98 kV/cm
5 mM	1.1 kV/cm	5 mM	0.92 kV/cm

The electric field amplitude required to kill 50% of cells (E_50_) was extracted for calcium concentrations of 0 mM, 1 mM, 3 mM, and 5 mM from the fitting functions reported in Fig 3. The relation between E_50_ and calcium concentration is shown. MATLAB software was used for calculation, n = 6.

### Confocal fluorescence imaging of ATP and permeability

**Fig 4:**
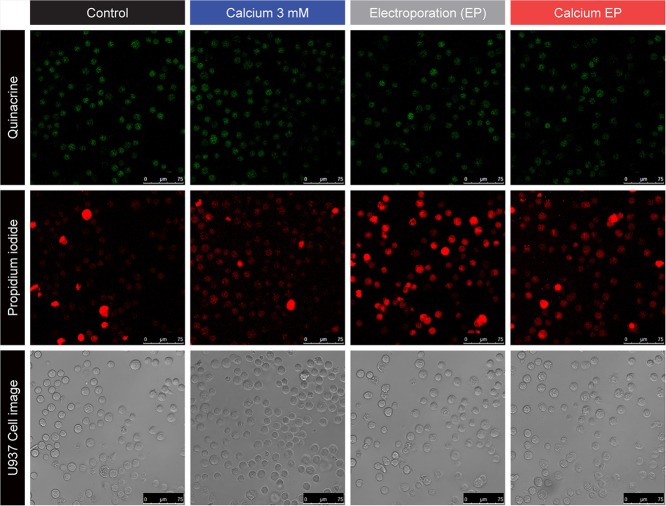
Scanning confocal images of U937 15 min after calcium electroporation. U937, a human leukaemia cell line. Final extracellular calcium chloride concentrations of 0 mM or 3 mM and applied electric field of 1.0 kV/cm. Quinacrine fluorescence (top row), 10 μM; propidium iodide fluorescence (middle row), 7.5 μM; phase contrast cell images (bottom row).

**Fig 5:**
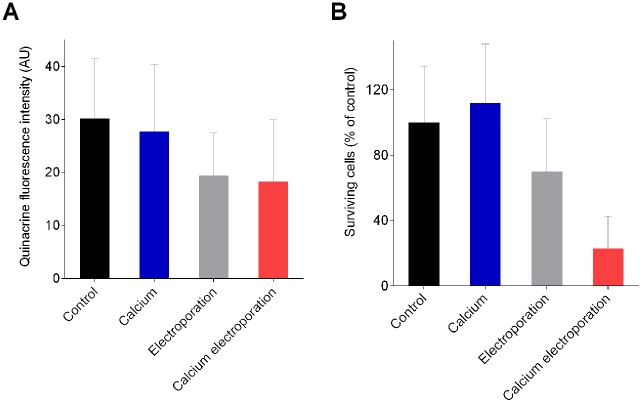
U937 cell response to calcium electroporation. U937, a human leukaemia cell line. (**A**) Cellular quinacrine fluorescence intensity of U937 15 min after calcium electroporation. The extracellular quinacrine concentration was 10 μM during loading and electroporation. Final extracellular calcium concentrations of 0 mM or 3 mM and applied electric field of 1.0 kV/cm were used. Columns depict the average of 180 cells (9 experiments × 20 cells). Arbitrary units (AU), mean + S.D., n = 9. (**B**) U937 cell viability measured 24 h after calcium electroporation. Viability assessed for U937 using resazurin cell proliferation assay. Extracellular calcium concentrations of 0 or 3 mM. Applied electric field of 1.0 kV/cm. Results are illustrated as percentage of control (no electroporation, no added calcium), mean + S.D., n = 6.

We used confocal fluorescence imaging of quinacrine-labelled cells to investigate changes in intracellular levels and distribution of ATP [27–30]. Fig 4 shows selected representative images taken 15 minutes after treatment. The average quinacrine fluorescence of calcium electroporated samples was about 30% lower than in the calcium-only samples (p<0.0001), schematically shown in Fig 5A. The electric field needed to kill half the cells (E_50_) under the explored conditions in presence of 3 mM calcium, was estimated at 0.70 kV/cm.

Permeabilisation of U937 cells marked by influx of propidium iodide was delayed and more irregular after calcium electroporation compared to electroporation without calcium (Fig 4).

We also observed morphological changes in calcium electroporated cells erratically exhibiting compromised membrane integrity, blebbing, shrinkage, or irregular cell shape (Fig 4).

### Resazurin viability assay

The results of the viability assay (Fig 5B) of U937 cells show that there were more viable cells in the sample treated with calcium alone than in the calcium-electroporated sample (p<0.0001).

## Discussion

This study explores the response in cellular ATP level and viability to different electroporation parameters and calcium concentrations for calcium electroporation treatment. Both the H69 and SW780 cell lines displayed a dose-dependent decrease in cellular ATP and viability with increasing electric field along with increasing calcium concentration (Fig 1A and 1B). The results do not fully uncover whether treatment can be optimized according to individual cell line sensitivity. The results showed that the H69 cell line required a high electric field to induce maximum ATP depletion (Fig 1A) and cell-kill (Fig 2A).

The difference between the responses of the two cell lines to the explored parameters for calcium concentration and electric field may partially be explained by difference in cell size. The cellular response to calcium electroporation is less dependent on cell size *in vivo* because of a general lack of extracellular space, affecting the cells ability to "hide" from pulse exposure [31], a reason that *in vitro* results on thresholds may not necessarily predict in vivo efficacy.

Calcium electroporation caused a significant short-term decrease in quinacrine fluorescence in U937 cells, which may indicate ATP depletion, and the treatment significantly decreased cell viability after 24 hours. More systematic investigations to follow could use an ATP-specific assay, such as luminescence from luciferin-luciferase.

Irreversible electroporation (IRE) is a procedure where cells are exposed to applied electric field parameters strong enough to induce permanent damage of the cell membrane, ultimately leading to disrupted ion homeostasis and cell death [14]. IRE causes tissue ablation with lower treatment efficiency in the margin of the targeted tissue where the electric field intensity is lowest. The ability to obtain higher cell kill and cause tissue ablation when increasing the electric field is the background for using IRE for cancer treatment [13], and is currently used for treatment of renal [32], hepatic [33] and pancreatic tumours [34], and potentially for prostatic [35] and intracranial tumours [36]. We have found that increasing the extracellular calcium concentration during electroporation induces greater cell kill, which implies that adding calcium, even in low doses, could potentially improve the therapeutic effect of IRE in lower intensity regions of targeted tissue, as the results in Table 1 demonstrate. Therefore, adding calcium to the IRE procedure could potentially increase the treatment radius and efficacy.

IRE and electroporation used in combination with chemotherapeutic drugs is gaining general acknowledgement as a treatment modality for solid tumours, and is used as palliative treatment for patients unfit for surgery (NICE clinical guideline 446, March 2013 [2,9–11]). Calcium electroporation has proven comparable in effectiveness to electrochemotherapy *in vitro* [7] and is currently undergoing the first non-inferiority clinical trial for the treatment of cutaneous metastasis (ClinicalTrials.gov-ID NCT01941901). The efficiency of calium electroporation as a novel cancer treatment modality is also being investigated for treatment of tumours in internal organs [37,38].

Previous studies investigating calcium electroporation have shown that the treatment affects cellular ATP and viability [6]. This study corroborates that theory with a demonstration of dose-dependency, and further supports the use of calcium as an efficient and inexpensive alternative to chemotherapeutics in electroporation procedures.

To our knowledge the relationship between calcium concentration and electric field in calcium electroporation treatment of cancer cells has not previously been investigated. Our data shows that calcium electroporation is effective with reduced electric fields if the calcium concentration is increased. More research is needed to fully understand how this novel cancer treatment modality induces cell death and the role that ATP depletion and disruption of the calcium homeostasis plays in the therapeutic effect of calcium electroporation.

## Conclusions

The effect of calcium electroporation is influenced by individual cell line responses and depends both on calcium concentration and on electric field amplitude. The study demonstrates that calcium electroporation significantly reduces cellular ATP and cancer cell survival. The results further support the use of calcium electroporation as a therapeutic modality in treatment of cancer as well as the possibility of lowering the applied electric field in future trials with electroporation.
