# Correction: Dose-Dependent ATP Depletion and Cancer Cell Death following Calcium Electroporation, Relative Effect of Calcium Concentration and Electric Field Strength

**DOI:** 10.1371/journal.pone.0128034

**Published:** 2015-05-07

**Authors:** Emilie Louise Hansen, Esin Bengisu Sozer, Stefania Romeo, Stine Krog Frandsen, P. Thomas Vernier, Julie Gehl

Fig 3 is incorrect. Please see the correct Fig 3 here.

**Fig 3:**
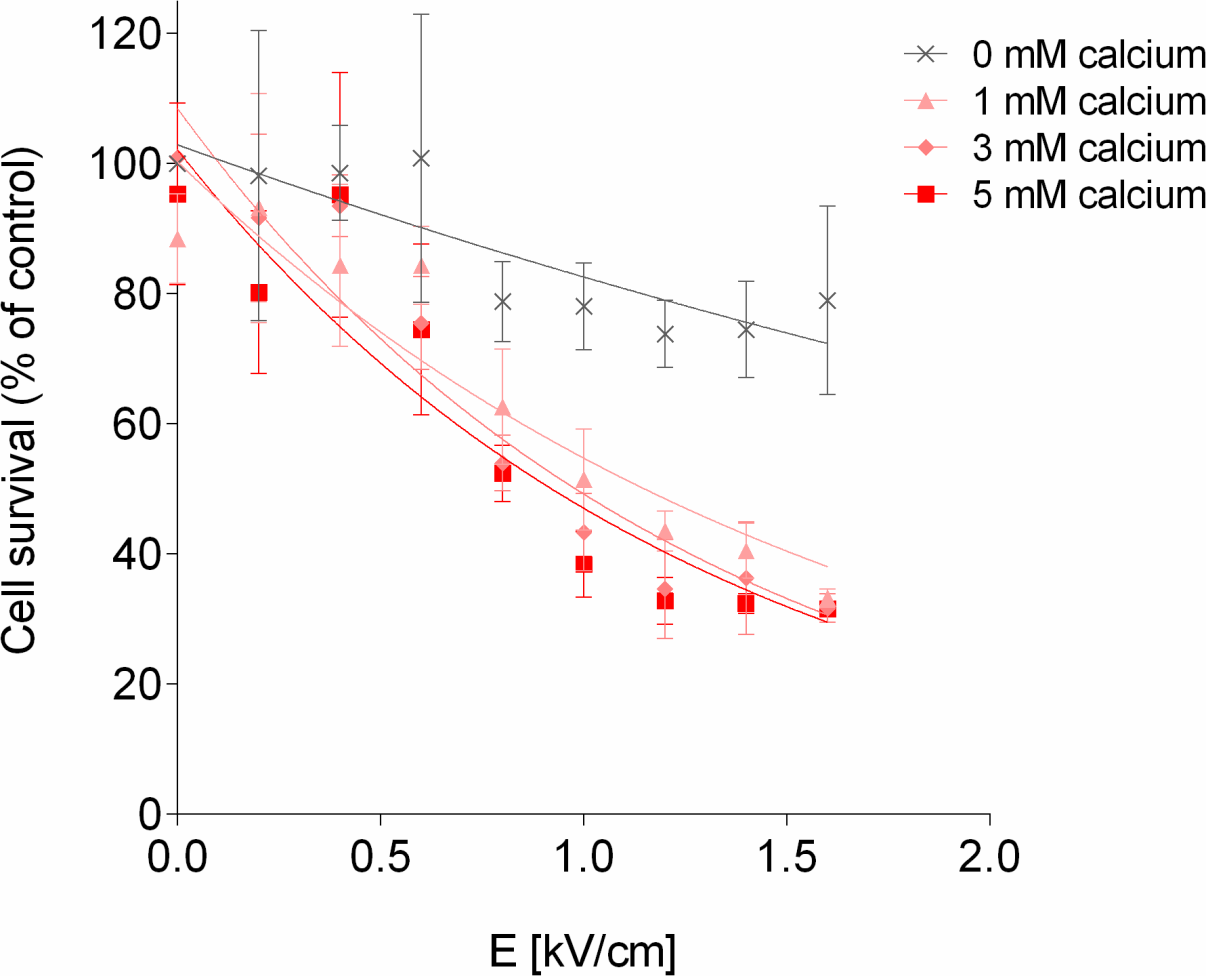
SW780 viability as a function of pulsed electric field and extracellular calcium concentration. Cell survival (%) versus electric field (E) at calcium electroporation using 0, 1, 3, or 5mM calcium in SW780 human bladder cancer cells assessed using MTS assay 24 hours after treatment. Electric field amplitude of 0.8 kV/cm, 1.0 kV/cm, 1.2 kV/cm, 1.4 kV/cm, or 1.6 kV/cm was applied. Fitting curves were derived using MATLAB software. Results are illustrated as percentage of control (no electroporation, no added calcium), mean ± S.D., n = 6.
